# A Treatise on Therapeutics and Pharmacology, or Materia Medica

**Published:** 1857-01

**Authors:** 


ART. VIII. *A Treatise on Therapeutics and Pharmacology, or Materia Medica.* By GEORGE B. WOOD, M.D., Professor of Medicine in the University of Pennsylvania, &c. In 2 vols. 8vo. J. B. Lippincott & Co. Philadelphia, 1856.

As a large majority of our subscribers have been already treated to an extended notice of the above valuable work in the last number of the Examiner, we deem it unnecessary to say more here than repeat the favorable opinion therein expressed. No recent American medical author has won a wider, better, and more deserved reputation than Dr. Wood; and the very flattering reception that the present fruit of his labor has received from the medical periodicals and the profession, both of this country and Great Britain, are sufficient evidence of its intrinsic merits. A few more productions of corresponding value, upon some of the other branches of medicine, from the pens of our native observers, would leave our medical schools no excuse for recommending foreign works to their students, and effectually remove the cause of the reproach so often cast upon us by European periodicals in reference to the paucity of our standard medical literature. To those of our readers who have not seen the review above referred to, we will only say, in the words of the London Lancet, "The same great principles of pathology pervade the present work as are found in the author's treatise on the Practice of Medicine, and which constitute the basis of what belongs to the general subject of therapeutics. We would strongly recommend Dr. Wood's volumes to the notice of the profession in this country. They will be found of great value to the student as well as the teacher, and are free from those technicalities which so much encumber works on materia medica of the present day."

